# 3D Winding Number: Theory and Application to Medical Imaging

**DOI:** 10.1155/2011/516942

**Published:** 2011-01-12

**Authors:** Alessandro Becciu, Andrea Fuster, Mark Pottek, Bart van den Heuvel, Bart ter Haar Romeny, Hans van Assen

**Affiliations:** ^1^Department of Biomedical Engineering, Eindhoven University of Technology, 5600 MB Eindhoven, The Netherlands; ^2^Department of Biology, Kaiserslautern University of Technology, 67653 Kaiserslautern, Germany

## Abstract

We develop a new formulation, mathematically elegant, to detect critical points of 3D scalar images. It is based on a topological number, which is the generalization to three dimensions of the 2D winding number. We illustrate our method by considering three different biomedical applications, namely, detection and counting of ovarian follicles and neuronal cells and estimation of cardiac motion from tagged MR images. Qualitative and quantitative evaluation emphasizes the reliability of the results.

## 1. Introduction

Critical points are very helpful for different purposes and applications in computer vision as key points, landmark points, anchor points, and others. In segmentation, for example, critical points have been used to characterize deforming areas of the brain [1] or to enhance ridges and valleys in MR images [2]. In image matching, mappings between the considered images are computed based on their critical points [3, 4]. Image matching has been also performed through the so-called *top points*, critical points for which the determinant of the Hessian matrix is equal to zero [5, 6], or through the popular Harris points [7] and the SIFT keypoint detector [8]. Critical points have also been used in motion estimation algorithms, where the optic flow field is generated from a sparse set of velocities associated to multi-scale anchor points [9, 10].

Critical point detection is an established research field. Blom [11], for example, classifies critical points by counting the sign changes between the analyzed pixels and its neighbors in a hexagonal grid. Nackman [12] defines the image topology in terms of slope districts. The ridge and valley lines are described as the ascending and descending slopes coming from saddle points. The dales and hills are identified as districts whose lines of slope converge to/come from the same pit/peak. These methods have been extensively employed for 2-dimensional applications. In recent years, there has been a strong increase of computational power, and 3D scalar images are becoming the standard data of investigation, especially in medical imaging. Three-dimensional critical point techniques allow for a more realistic analysis of human organ behavior. For example, tracking algorithms applied on a 2-dimensional heart image sequence retrieve only in-plane contractions and rotations of the cardiac walls but miss the through-plane components. The through-plane components are instead retrieved with 3-dimensional optic flow approaches. In this paper, we show an application where the presented critical point detection algorithm is embedded in a feature point-based motion estimation technique.

In this paper, we work with a topological number (from homotopy theory) that can locate critical points of scalar images in an arbitrary number of dimensions. In two dimensions, it reduces to the so-called winding number and has been studied in detail in [13–15]. In physics, and in modern cosmology in particular, the winding number appears in the context of topological defects such as monopoles, cosmic strings, and domain walls (see, e.g., [16] and references therein). We consider this topological number in three dimensions and refer to it as 3D winding number. Properties of this approach are significant.

- The 3D winding number provides information on the character of the critical points.
- The winding number is independent of the shape of hypersurface S around which it is calculated. It is a topological entity.

The paper is organized in the following way. After some preliminaries (Section 2.1), we treat extensively the theoretical aspects of the winding number in three dimensions and explain the implementation of our algorithm (Sections 2.2 and 2.3). In Sections 2.4 and 2.5, we describe a methodology to refine the position of the retrieved critical points, and we propose a classification of critical points based on the winding number. Furthermore, we test the viability of our method by considering three different biomedical applications, namely, follicle and neuronal cell counting and cardiac motion estimation in Sections 3.1, 3.2, and 3.3, respectively. Finally, in Section 4, we discuss the results and possibilities for future work.

## 2. Theory

### 2.1. Preliminaries

A critical point of a smooth function *f*(*x*^1^,…, *x*^*n*^) is a point **x** = (*x*^1^,…, *x*^*n*^) for which the gradient of *f* vanishes, ∇*f*|_*x*_ = 0. In any other case, the point is said to be regular. Critical points can be further classified depending on whether the Hessian matrix at the considered point is singular:

(1) $${\det\;\;\left(\partial_{i}\partial_{j}f\right)|}_{x}=0.$$

This is obviously the case if one or more matrix eigenvalues are zero. Such critical points are called degenerate. Otherwise, we deal with nondegenerate critical points. 

We are interested in finding and classifying critical points of a scalar image *L*(**x**). We will do so by computing a topological quantity *ν* at every point in the image. The topological number of a *d*-dimensional scalar image at a point **x** (with at most isolated singularities) is defined by [13]

(2) $$\nu =\oint_{S}\Phi \left(x\right),$$

where Φ is a (*d* − 1)-form depending on the image intensity and its derivatives (see, e.g., [17] for a general discussion of differential forms). The precise definition of Φ in *d* dimensions can be found in [13]. In this paper, we will only consider the case *d* = 3 (further details are given in the next section). The integration is performed on a closed, oriented (hyper) surface *S* around the considered point.

An important property of Φ is the fact that it is a closed form, *d*Φ = 0. If the image has no singularities in the region *V* enclosed by *S*, the generalized Stoke's theorem can be applied to (2):

(3) $$\nu =\oint_{S}\Phi \left(x\right)=\int_{V}d\Phi \left(x\right)\equiv 0.$$

Therefore, the quantity *ν* is just zero at a regular point. At a singular point, it takes values of *kπ*, with *k* some nonzero integer number depending on the number of dimensions and the character of the singularity. (This is true for *d* ≥ 2.) 

The described number is called topological because it does not depend on the chosen hypersurface of integration in (2). Another important property is the fact that it is conserved within such a hypersurface; that is, when two or more singularities are enclosed, their topological numbers add up. We refer to [13] for a more detailed discussion on these and other properties of *ν* in an arbitrary number of dimensions.

### 2.2. Winding Number in Three Dimensions

 In three dimensions, the integrand in equation (2) is a 2-form given by [13]:

(4) $$\Phi =\frac{L_{i}dL_{j}\wedge dL_{k}\epsilon^{ijk}}{{\left(L_{l}L_{l}\right)}^{3/2}},\;i,j,k,l=x,y,z.$$

Here, the indices *i*, *j*, *k*, *l* can take on values *x*, *y*, or *z*, *L* = *L*(*x*, *y*, *z*) is the intensity function of a 3-dimensional image, *L*_*x*_, *L*_*y*_, *L*_*z*_ are the components of the spatial gradient of the intensity function, ∇*L* = (*L*_*x*_, *L*_*y*_, *L*_*z*_), and *ϵ* is the 3-dimensional Levi-Civita symbol. The wedge product is represented by ∧. In this paper, we use Einstein's summation convention; that is, a sum is taken over repeated indices appearing in both subscripts and superscripts. In explicit form, (4) reads

(5) $$\Phi =\frac{2}{{||\nabla L||}^{3}}\left(L_{x}dL_{y}\wedge dL_{z}+L_{y}dL_{z}\wedge dL_{x}+L_{z}dL_{x}\wedge dL_{y}\right),$$

where ||∇*L*|| is the gradient norm. Using the following relations:

(6) $$dL_{i}=L_{ix}dx+L_{iy}dy+L_{iz}dz,$$

we can rewrite (5) as

(7) $$\begin{array}{cc} \Phi = & \frac{2}{{||\nabla L||}^{3}}\{dx\wedge dy[\left(L_{yx}L_{zy}-L_{yy}L_{zx}\right)L_{x}\\ & \;\;\;\;\;\;\;+\left(L_{zx}L_{xy}-L_{zy}L_{xx}\right)L_{y}\\ & \;\;\;\;\;\;\;+\left(L_{xx}L_{yy}-L_{xy}L_{yx}\right)L_{z}]\\ & \;\;\;+dy\wedge dz[\left(L_{yy}L_{zz}-L_{yz}L_{zy}\right)L_{x}\\ & \;\;\;\;\;\;\;+\left(L_{zy}L_{xz}-L_{zz}L_{xy}\right)L_{y}\\ & \;\;\;\;\;\;\;+\left(L_{xy}L_{yz}-L_{xz}L_{yy}\right)L_{z}]\\ & \;\;\;+dz\wedge dx[\left(L_{yz}L_{zx}-L_{yx}L_{zz}\right)L_{x}\\ & \;\;\;\;\;\;\;+\left(L_{zz}L_{xx}-L_{zx}L_{xz}\right)L_{y}\\ & \;\;\;\;\;\;\;+\left(L_{xz}L_{yx}-L_{xx}L_{yz}\right)L_{z}]\}. \end{array}$$

This expression was also given in [18]. After further inspection, we notice that it can be reformulated in the following way:

(8) $$\begin{array}{cc} \Phi = & \frac{2}{{||\nabla L||}^{3}}\{dx\wedge dy\left[\left(\nabla L_{x}\times \nabla L_{y}\right)\cdot \nabla L\right]\\ & \;\;\;\;\;+dy\wedge dz\left[\left(\nabla L_{y}\times \nabla L_{z}\right)\cdot \nabla L\right]\\ & \;\;\;\;\;+dz\wedge dx\left[\left(\nabla L_{z}\times \nabla L_{x}\right)\cdot \nabla L\right]\}, \end{array}$$

where ∇*L* = (*L*_*x*_, *L*_*y*_, *L*_*z*_) and

(9) $$\nabla L_{x}\equiv \partial_{x}\left(\nabla L\right)=\left(L_{xx},L_{yx},L_{zx}\right),$$

∇*L*_*y*_, ∇*L*_*z*_ are defined analogously. This new form is more elegant and simpler to work with. Comparing (7) and (8) it is also clear that the latter form will be easier to implement. In what follows, we will therefore use expression (8) rather than (7). In compact form, we have

(10) $$\Phi =\frac{1}{{||\nabla L||}^{3}}\left(\nabla L_{i}\times \nabla L_{j}\right)\cdot \nabla Ldx^{i}\wedge dx^{j},$$

where *i*, *j* take on values *x*, *y*, or *z*. In this formulation, it is trivial to check that Φ is antisymmetric as the vector product is anticommutative.

### 2.3. Implementation

We study the nature of every voxel by performing the integration of expression (8) on a 3 × 3 × 3 cube that contains it. Note that, for each face of the cube, only one term in (8) survives in the integration given by (2). For example, if we integrate on a cube face with *z* = constant, it is clear that *dz* = 0, and therefore only the first term has to be taken into account. 

One of the issues we face in the implementation is the integration of differential forms. We make use of the following identity for integration of differential forms in Euclidean space [19]:

(11) $$\begin{array}{cc} & \int_{\Omega }f\left(x^{1},\ldots ,x^{n}\right)dx^{1}\wedge \cdots \wedge dx^{n}\\ & \;\;=\pm \int_{\Omega }f\left(x^{1},\ldots ,x^{n}\right)dx^{1}\cdots dx^{n}. \end{array}$$

Here, *f*(*x*^1^,…, *x*^*n*^) *dx*^1^∧⋯∧*dx*^*n*^ is an *n*-form in ℝ^*n*^ and *Ω* is an oriented domain. (If the considered differential form has more than one component the identity simply holds for each one of them.) Note that the integral on the right-hand side is just the usual integral of the function *f*(*x*^1^,…, *x*^*n*^). The sign on the right-hand side depends on the orientation of the considered integration domain (+ for positively oriented, − for negatively oriented). For example, from (8) and (11), the integration on *z* = constant opposite cube faces reads

(12) $$\begin{array}{cc} \nu_{xy} & =\int_{z=\text{const}.}\Phi \\ & =\frac{2}{{||\nabla L||}^{3}}(\int_{\text{up}}\left(\nabla L_{x}\times \nabla L_{y}\right)\cdot \nabla Ldxdy\\ & \;-\int_{\text{down}}\left(\nabla L_{x}\times \nabla L_{y}\right)\cdot \nabla Ldxdy). \end{array}$$

We consider the image intensity function on the faces of a 3 × 3 × 3 cube to be *L* = *L*(*x*_*α*+*a*_, *y*_*β*+*b*_, *z*_*γ*+*c*_), where *a*, *b*, *c* are shifting indices of a plane on the cube taking on values 0, 1, 2 and *α* = 1,…, *NB*_*x*_ − 2, *β* = 1,…, *NB*_*y*_ − 2, *γ* = 1,…, *NB*_*z*_ − 2 are indices of the image volume with *NB*_*x*_, *NB*_*y*_, and *NB*_*z*_ representing the volume size in *x*, *y*, and *z* directions. With these conventions, equation (12) can be expressed numerically as

(13) $$\begin{array}{cc} \nu_{xy}^{\alpha ,\beta ,\gamma } & =\overset{2}{\underset{a,b=0}{\sum }}\left(\nabla L_{x}\times \nabla L_{y}\right)\cdot \nabla L\left(x_{\alpha +a},y_{\beta +b},z_{\gamma +2}\right)\\ & \;-\overset{2}{\underset{a,b=0}{\sum }}\left(\nabla L_{x}\times \nabla L_{y}\right)\cdot \nabla L\left(x_{\alpha +a},y_{\beta +b},z_{\gamma }\right). \end{array}$$

The winding numbers on planes *x* = constant and *y* = constant can be computed in a similar way. The total winding number for the considered cube is then

(14) $$\nu^{\alpha ,\beta ,\gamma }=\nu_{xy}^{\alpha ,\beta ,\gamma }+\nu_{yz}^{\alpha ,\beta ,\gamma }+\nu_{zx}^{\alpha ,\beta ,\gamma }.$$

The numerical implementation of the 3D winding number algorithm can be summarized in the following steps.

- Load scalar image *L*(*x*, *y*, *z*).
- Calculate the winding number for all voxels in the image volume

**for** *α* = 1 to *NB*_*x*_ − 2 **do**
 **for** *β* = 1 to *NB*_*y*_ − 2 **do**
  **for** *γ* = 1 to *NB*_*z*_ − 2 **do**
   *ν*^*α*,*β*,*γ*^ = *ν*_*xy*_^*α*,*β*,*γ*^ + *ν*_*yz*_^*α*,*β*,*γ*^ + *ν*_*zx*_^*α*,*β*,*γ*^
  **end for**
 **end for**

**end for**
- Divide the outcomes of *ν*^*α*,*β*,*γ*^ by 4*π*.
- In order to distinguish the type of critical points retrieved (maxima or minima from saddles), extract the sign of the Hessian matrix determinant at locations, where *ν*^*α*,*β*,*γ*^ ≠ 0.

### 2.4. Refinement of Critical Point Positions

**Figure 1:**
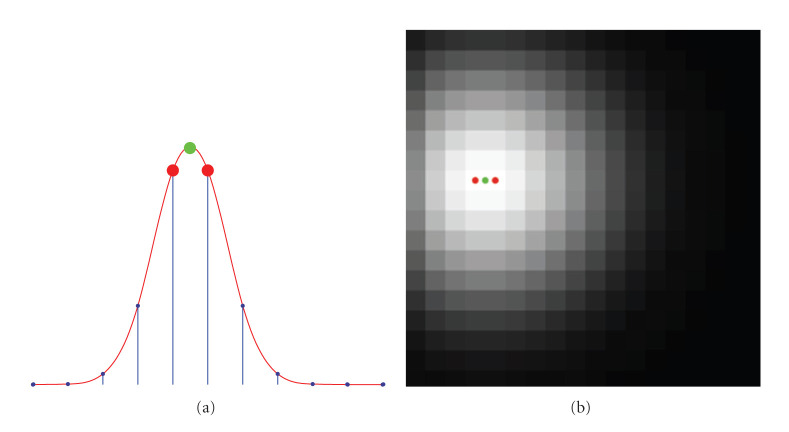
Critical point refinement. (a) A continuum Gaussian signal in 1 dimension and the corresponding sampled signal. The sampled signal shows maxima at two nearby positions (points in red), which are at different locations from the real maximum (point in green). (b) Rasterized version of a 2-dimensional Gaussian signal. Red points are the retrieved maxima, whereas the green point is the true maximum obtained after the refinement.

 Due to signal discretization, the retrieved critical point location might not be completely accurate (see Figure 1 for an illustration of this issue for the 1D and 2D case). The position can be refined at subpixel level by considering the Taylor expansion of the intensity gradient around the retrieved point:

(15) $$\begin{array}{cc} \nabla L\left(x\right) & =(L_{x}\left(x_{e}\right)+\left(x-x_{e}\right)L_{xx}\left(x_{e}\right)+\left(y-y_{e}\right)L_{xy}\left(x_{e}\right)\\ & \;\;+\left(z-z_{e}\right)L_{xz}\left(x_{e}\right),\\ & \;L_{y}\left(x_{e}\right)+\left(x-x_{e}\right)L_{yx}\left(x_{e}\right)+\left(y-y_{e}\right)L_{yy}\left(x_{e}\right)\\ & \;\;+\left(z-z_{e}\right)L_{yz}\left(x_{e}\right),\\ & \;L_{z}\left(x_{e}\right)+\left(x-x_{e}\right)L_{zx}\left(x_{e}\right)+\left(y-y_{e}\right)L_{zy}\left(x_{e}\right)\;\\ & \;\;+\left(z-z_{e}\right)L_{zz}\left(x_{e}\right)), \end{array}$$

where **x** = (*x*, *y*, *z*) and **x**_*e*_ = (*x*_*e*_, *y*_*e*_, *z*_*e*_) denote the true and estimated critical point locations, respectively. We can write equation (15) in a more compact form:

(16) $$L_{i}\left(x\right)=L_{i}\left(x_{e}\right)+\left(j-j_{e}\right)L_{ij}\left(x_{e}\right),$$

where *i*, *j* can take on values *x*, *y*, or *z*. The intensity gradient at a critical point vanishes. The refined critical point position is therefore

(17) $$\left(\begin{array}{c} x\\ y\\ z \end{array}\right)=\left(\begin{array}{c} x_{e}\\ y_{e}\\ z_{e} \end{array}\right)-H^{-1}\left(x_{e}\right)\left(\begin{array}{c} L_{x}\left(x_{e}\right)\\ L_{y}\left(x_{e}\right)\\ L_{z}\left(x_{e}\right) \end{array}\right).$$

Here, *H* is the Hessian matrix. Equation (17) provides the critical point position at subpixel level and can be iterated until the desired accuracy has been reached. 

### 2.5. Classification of Critical Points

 In three dimensions, there are four types of nondegenerate critical points, namely, minima, 1-saddles, 2-saddles, and maxima. They are characterized by the number of negative eigenvalues of the 3 × 3 Hessian matrix, the index, at the corresponding point: 0, 1, 2, or 3. Each 1-saddle (2-saddle) point is connected to two, not necessarily distinct, minima (maxima) by integral lines. A more detailed description of 3D saddle points can be found in [20]. 

The winding number at a certain image point is given by the integral of expression (10) on an appropriate surface enclosing the point. The winding number of (isolated) critical points in three dimensions takes values of 4 *kπ*, with *k* = ±1 [18, 21]. We will argue that the winding number can be used for classification of extrema and saddle points in 3D. As a matter of fact, the winding number is able to distinguish between the two types of saddle points in 3D. 

 In Tables 1 and 2, we summarize the explicit values for the index and winding number of the different types of critical points. For completeness, we treat also the 2-dimensional case. Note that extrema in 3D can have either positive or negative winding number, unlike the 2D case. Saddles have positive or negative winding number as well, depending on the type of saddle point. It is now possible to classify critical points according to their winding number. Once the sign has been calculated, it suffices to examine the image intensity at the considered point and its close neighborhood to distinguish between a minimum and a 2-saddle or a maximum and a 1-saddle.

The proposed correspondence between the index and winding number in three dimensions is well grounded. The following has been shown for a nondegenerate critical point in an arbitrary number of dimensions [13]:

(18) $$\nu =\mathrm{sign}\;\left(\det\;\;H\right)C_{d},$$

where *H* is the Hessian matrix and *C*_*d*_ is a constant depending only on the number of dimensions *d*. In three dimensions, *C*_*d*_ is equal to 4*π*. The relation between the winding number and the sign of the Hessian in *d* = 3 is given in Table 3. This is clearly in agreement with the postulated winding number for the different types of critical points.

## 3. Experiments

The proposed algorithm has been implemented in Mathematica [22], and it has been tested on three different biomedical applications, namely, follicle detection, neuronal cell counting, and cardiac left ventricle motion analysis. In order to perform the experiments we make use of the scale-space framework [21, 23–26]. The Gaussian scale-space representation *L* : ℝ^3^ × ℝ^+^ of a 3-dimensional static image **x** ↦ *f*(**x**) ∈ *𝕃*_2_(ℝ^3^) is given by the spatial convolution with a Gaussian kernel

(19) $$L\left(x,s\right)=\left(f*\phi_{s}\right)\left(x\right)\;\text{with}\;\phi_{s}\left(x\right)=\frac{1}{4\pi s}\exp\;\;\left(-\frac{x^{2}}{4s}\right),$$

where **x** = (*x*, *y*, *z*) ∈ ℝ^3^ and *s* > 0 represents the scale. In the remainder of the paper, the image intensity function should be regarded as a function of both location and scale, *L* = *L*(**x**, *s*).

### 3.1. Follicle Detection

**Figure 2:**
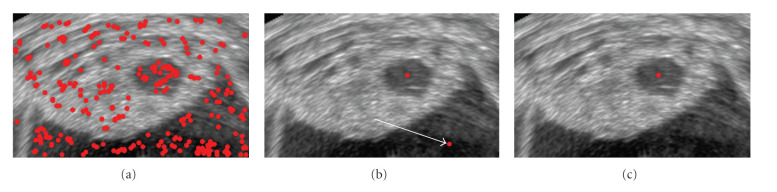
Follicle detection. The red dots highlight the detected minima. (a) The image shows detected minima at scale *s* = 2. The image is very noisy, and the algorithm detects also the minima corresponding to noisy grains (false positives). (b) The image shows minima detected at scale *s* = 9. The arrow shows a minimum detected outside the ovarian tissue (false positive), whereas the red dot inside the ovarian tissue corresponds to the center of a follicle. (c) In this image, the false positive outside the ovarian boundaries has been filtered out.

**Figure 3:**
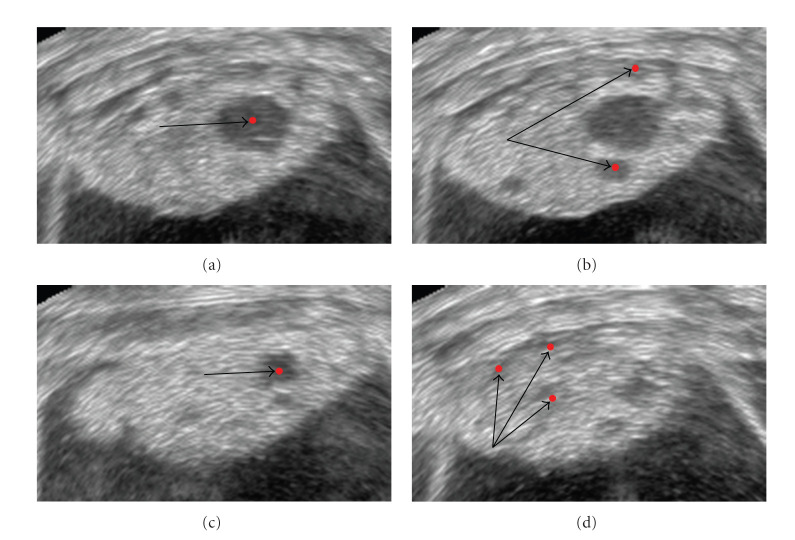
Follicle detection. 2D slices of the 3D ultrasound image smoothed data set corresponding to one of the patients. Lighter areas display the ovary; dark circular blobs are the follicles. Red dots indicate retrieved local minima in 3D at scale *s* = 9 voxels.

Ovarian follicles are the basic eggs of the female reproductive system. In particular, the number of primordial follicles decreases with the age reaching a minimum during the menopause. Therefore, follicle analysis and counting may provide information on fertility prospects [27–29]. At the stage of development that they can be measured with 3D ultrasound, the human follicles present roughly a spherical shape with a typical diameter of two to five *mm* and appear darker with respect to surrounding tissue on ultrasound images (see Figures 2 and 3) [30].

Detection and counting of follicles is usually carried out manually by inspecting the 2D slices from a 3D data set. This is a repetitive and tedious task which might introduce mistakes especially in the typically noisy data sets. Robust and automated detection of follicles is therefore useful.

In the experiments, we automatically locate and count ovarian follicles of three different patients using ultrasound image volumes with a size of 128 × 110 × 180, 138 × 116 × 176, and 180 × 108 × 126 voxels, respectively. Image acquisition has been carried out by an experienced echographer with 3D ultrasound system Combison 5600 (Kretz Technik AG, Medicor, Austria/Korea), which has been equipped with a 12 MHz transvaginal 3D probe of 2.2 cm. The system performs image volume acquisition in about 2 seconds and allows to reliably detect follicles with diameter of 3 mm or bigger. The image data were processed in order to include only the ovary after the scanning.

In the images, the center of the follicles exhibits a local minimum intensity. In these points, the intensity gradient vanishes. Due to the noisy nature of the images, the data sets exhibit several locations, where minima occur outside the follicle structure, producing false positives. The follicle detection algorithm consists of two main steps as follows.

- The 3D volume images were isotropically smoothed using different scales.
- Evaluation of the 3D winding number is carried out in order to retrieve the follicle centers.

In this procedure, we observe a tradeoff situation for the choice of the proper scale. We notice that follicles present a larger structure with respect to grains of the raw data. In the experiments, the scale is heuristically chosen sufficiently high to avoid grain detection (see Figure 2(a), for critical point detection at small scale), but not so high that smaller follicles are missed. In this experiment, the results of follicles extraction have been achieved at scale *s* = 9 voxels. The same critical point detection procedure has been followed also for the experiments on neuronal cell counting and cardiac motion estimation. 

After critical point localization, the ovarian tissue has been manually segmented in each slice in order to create a mask and filter out the minima retrieved outside the ovarian boundaries (false positives) (see Figures 2(b) and 2(c)). 

In the three data sets, results establish the presence of 19 follicles for patient one, 8 for patient two, and 11 for patient three. Manual counting of an expert revealed 17 follicles for patient one, 7 for patient two, and 10 for patient 3 ([18, page 68, Table 2, patient one, two, and three]). The computational time for each data set at scale *s* = 9 is less than 5 minutes on a PC with Intel Core 2 Duo 2 GHz processor and 4 GB RAM. The same computer has been used to carry out the experiments of neuronal counting and cardiac motion estimation. For each individual, the amount of detected follicles indicates relatively good fertility prospects according to [31], especially in the case of patient one. In Figures 2 and 3, retrieved minima are associated to red dots.

### 3.2. Neuronal Cell Counting in Cerebellum

The cerebellum is a region of the central nervous system located in the so-called hindbrain. It is responsible for motor activity and regulation of muscle tone and also plays an important role in cognitive and language functions in humans. In spite of occupying only around ten per cent of the whole brain volume, the cerebellum contains about fifty percent of all neurons. The number of neurons varies depending on the age and health condition, such as in Alzheimer's disease [32]. Cell density is useful biomarker; however, neuronal cell counting is often done manually. This is a time-consuming task, where human mistakes cannot be excluded. The eye of the observer will perform increasingly worse at such repetitive tasks. As a result, estimations made for large number of cells may become unreliable. For example, the number of Purkinje cells (the principal neurons of the cerebellum) in humans has been estimated to be between 14 and 26 millions [33]. Automatic counting methods are therefore preferable. 

Several cell counting methods can be found in the literature. They are mostly based on the cell density distribution in a certain volume and a good guess of the scientist [33–38]. These methods assume that the cell distribution in the volume of reference stays uniform in the whole region of interest. If this is not the case, such methods will not provide a reliable outcome. The algorithm proposed in this paper carries out automatic detection and counting without any assumptions about the cell distribution. Therefore, it may overcome the shortcomings of such techniques and provide more accurate results. 

**Figure 4:**
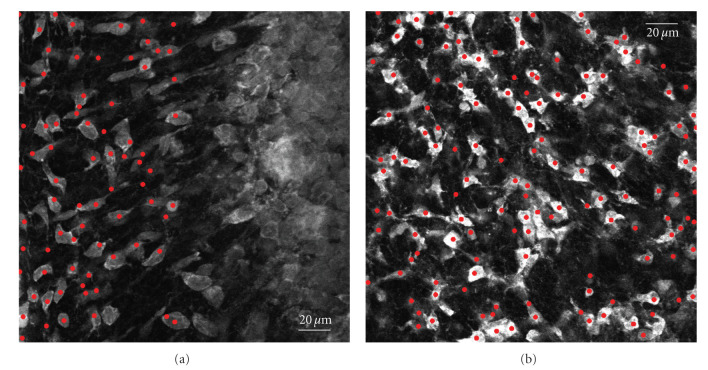
Cerebellum cell counting. (a) A slice of stack 1. (b) A slice of stack 2. Red dots indicate neurons retrieved by the algorithm.

In the experiments, we consider two image volumes of neurons labeled with propidium iodide with dimensions 2048 × 2048 × 25 (164.5 × 164.5 × 42.7 *μ*m) and 2048 × 2048 × 15 (230.3 × 230.3 × 32 *μ*m) voxels, respectively. The images were acquired with a confocal microscope. They correspond to two different regions of an 18-day-old rat cerebellum. The neuron cell bodies are seen to be roughly spherical (see Figure 4). Part of the first image volume shows dense labeling which could not be discriminated into single cells (see right-hand side of Figure 4(a)). As a consequence we could not investigate the whole volume. 

Neurons have been retrieved as local minima with the proposed algorithm using scale 9 voxels, after enhancing the blobs in the image volume using a scale-normalized Laplacian operator. Our method retrieved 250 cells in stack 1 and 376 cells in stack 2 (Figure 4). A careful visual counting has been carried out on the first 8 slices of stack 1 by an expert neurobiologist, who could recognize 102 neuronal cells. Every slice was carefully inspected in order not to count the same cell twice and not to miss smaller cells closer to the bigger ones. This investigation took between 20 and 30 minutes. Although our method has not been optimized for speed purposes, it needed roughly 10–15 minutes to detect 112 neurons on the same data subset. 

Additionally, we compared the algorithm outcomes with the performance of a simple and fast technique based on the extraction of maxima and minima taking into account the local image intensity [21]. In this method, the intensity of each voxel is compared with the intensity of the respective 26 neighbors. Both approaches provided similar results: 250 cells and 376 cells for stacks 1 and 2 using the 3D winding number and 271 cells and 361 for stacks 1 and 2 using the critical point detector based on intensities.

### 3.3.  3D Cardiac Motion Estimation

**Figure 5:**
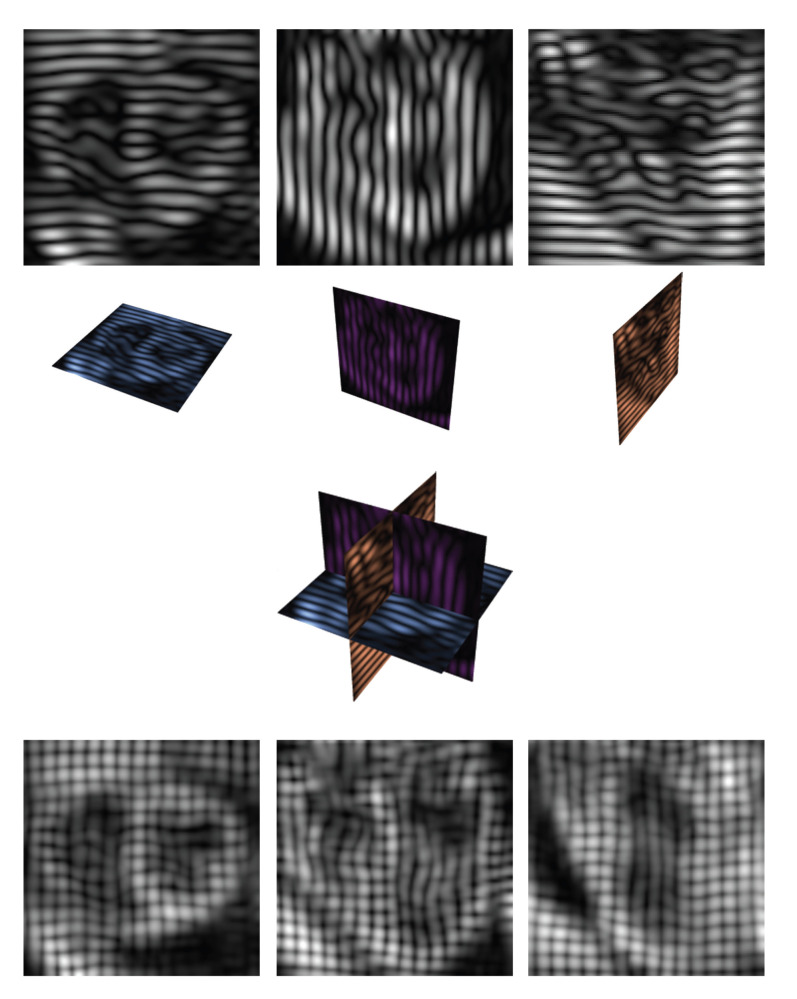
Cardiac tagged MR images, frame 3. Rows 1 and 2 from left to right: Short axis view with horizontal tags, 2 long axis views with vertical and horizontal tags, respectively. Row 3: Combination of the image planes. Row 4 displays the outcome of the combination of image planes. The images exhibit a chessboard pattern.

 Cardiac disease may strongly influence the dynamic behavior of the cardiac muscle. Estimation and visualization of the cardiac motion may become an important tool for diagnosis, providing indications of progress of the disease and/or therapy. Optic flow methods measure the apparent velocity of moving patterns in an image sequence. At the beginning of the 1980s, Horn and Schunck [39] introduced an optic flow approach based on brightness constancy, estimating the motion by solving the so-called Optic Flow Constraint Equation (OFCE). This technique, however, may not be the preferable choice for extracting motion from tagged MR images (see Figure 5 row 1). (The term tags refers to the sinusoidal pattern on the MR images, introduced with the goal to enhance the visualization of the tissue movement [40].) For these images, the constant intensity assumption does not hold due to tag fading under spin-lattice relaxation time (indicated by T1). 

 Over the years cardiac motion estimation has become a well-established research field. In the literature, however, there are few optic flow algorithms for 3D cardiac images [41, 42] due to the lack of data sets and sufficient available computational power in the past years. 

A 3D motion field exhibits expansions, contractions, and twistings of the cardiac tissue, making the results more realistic with respect to the ones provided by a 2D velocity field, where the through-plane motion component is missing. In the experiments, we investigate a 3D tagged MR image sequence of a human heart. Cardiac motion is estimated by calculating the velocity of critical points, maxima in this case, which are located at the tag crossings. This optic flow technique is not based on brightness conservation; therefore, it can be robustly applied directly on tagged MRI. In [43], a similar 3D motion estimation procedure has been presented. In this case, the critical points have been extracted by a methodology based on zerocrossings.

#### 3.3.1. Cardiac Image Data Set

The cardiac data used in the experiments consists of 23 frames with a temporal resolution of 30 ms, acquired by a 3D CSPAMM sequence [44]. Each frame presents 14 slices in the short axis and two different long axis views (Figure 5 row 1); the images display a size of 112 × 112 pixels, with 1 × 1 mm^2^ of pixel resolution. The recorded slices are perpendicular with respect to each other, and, in the experiments, we combine them to obtain a grid (Figure 5 rows 2, 3, and 4, resp.). Due to sparseness in the slices, we interpolate the 14 slices in each frame in order to obtain image voxels of 112 × 112 × 112 pixels.

#### 3.3.2. Calculation of Velocity at Critical Points Position

 As already mentioned, we are interested in tracking the critical points (maxima) that occur at the tag crossings of the chessboard-like pattern displayed in row 4 of Figure 5. In this case, we have a sequence of images, and therefore the image intensity is also a function of time, that is, *L*(**x**(*t*), *s*, *t*), where **x**(*t*) = (*x*(*t*), *y*(*t*), *z*(*t*)). The feature points move along with the cardiac tissue, since they are part of the tags. We also mentioned that MR tags fade due to relaxation time *T*_1_. This property does not influence the vanishing image gradient as long as the tags are visible, and therefore it does not affect the maxima detection at the tag crossings. 

By definition, the gradient of an image sequence *L*(**x**(*t*), *s*, *t*) vanishes at critical point positions

(20) ∇L(x(t),s,t)=0,

where ∇ denotes the spatial gradient and *s* and *t* represent the scale and time, respectively. In order to calculate the velocity at points with local maximum intensity (tag crossings) over time, we differentiate (20) with respect to time *t* and apply the chain rule for implicit functions. Hence, (21)

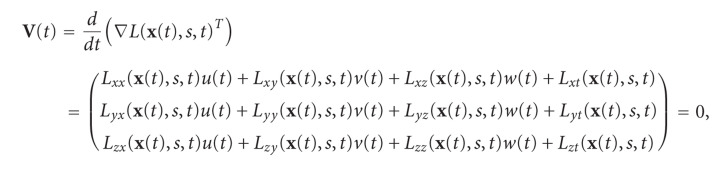

where *d*/*dt* is the total time derivative, *T* indicates transpose and *u*(*t*) = *dx*/*dt*, *v*(*t*) = *dy*/*dt*, and *w*(*t*) = *dz*/*dt* represent the velocity components in horizontal, vertical, and through-plane directions. In the experiments described in this section, we use a fixed scale *s* for all frames, for each experiment. Equation (21) can be reformulated in order to extract the velocities *u*, *v*, and *w*. Hence,

(22) $$V\left(t\right)=\left(\begin{array}{c} u\left(t\right)\\ v\left(t\right)\\ w\left(t\right) \end{array}\right)=-H{\left(x\left(t\right),s,t\right)}^{-1}\frac{\partial \left(\nabla L{\left(x\left(t\right),s,t\right)}^{T}\right)}{\partial t},$$

where *H* represents the spatial Hessian matrix of image *L*. In the literature, similar optic flow approaches that calculate velocity estimation at feature point location using the Hessian matrix are discussed in [9, 10, 43, 45].

#### 3.3.3. Experiments on 3D Image Sequences

**Figure 6:**
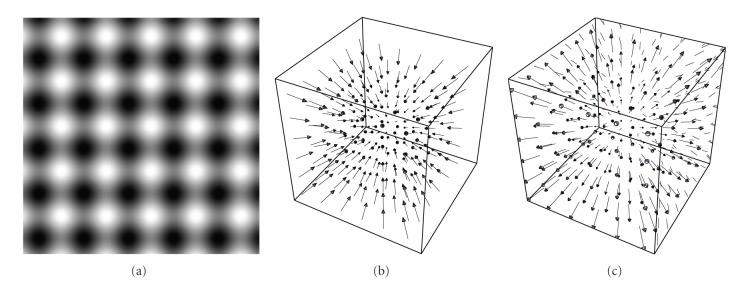
(a) The image shows a 2-dimensional slice of the 3-dimensional artificial phantom. (b) and (c) The images display the vector field of two successive frames of the phantom.

 In order to assess the accuracy of the extracted vector field, the motion algorithm has been tested on a sine phase grid artificial phantom (see Figure 6(a)) that exhibits contractions and expansions (see vector fields in Figures 6(b) and 6(c)). The phantom consists of 19 frames with resolution of 79 × 79 × 79 voxels and tags of 9 voxels wide. The ground truth velocity vector of the phantom is given by

(23) $$\begin{array}{cc} V_{\text{GT}}\left(t\right) & =\left(u_{\text{GT}},v_{\text{GT}},w_{\text{GT}}\right)\\ & =\frac{\left(m-2n\cdot t\right)}{\left(l+\left(m-n\cdot t\right)t\right)}\left(x-l,y-l,z-l\right), \end{array}$$

where *x*, *y*, *z*, *t* represent the spatial and temporal coordinates and *l*, *m*, *n* are constant parameters (related to the length of the vectors) set to 40, 4, and 0.2, respectively.

The accuracy has been described in terms of average angular error [46]:

(24) $$\begin{array}{cc} \text{AAE} & =\text{arccos}\;(\frac{V_{\text{GT}}\left(t\right)}{\sqrt{u_{\text{GT}}{\left(t\right)}^{2}+v_{\text{GT}}{\left(t\right)}^{2}+w_{\text{GT}}{\left(t\right)}^{2}}}\\ & \;\;\;\;\;\cdot \frac{V\left(t\right)}{\sqrt{u{\left(t\right)}^{2}+v{\left(t\right)}^{2}+w{\left(t\right)}^{2}}}). \end{array}$$

The motion estimation of the artificial phantom has been carried out from frame 8 to frame 11 in order to avoid outliers due to temporal boundary conditions and at scale *s* = 3.5 voxels. The computation of the optic flow field took roughly 5 to 10 minutes per frame. The average angular error is AAE = 2.68, degrees and the respective standard deviation (std) is std = 2.89 degrees. 

**Figure 7:**
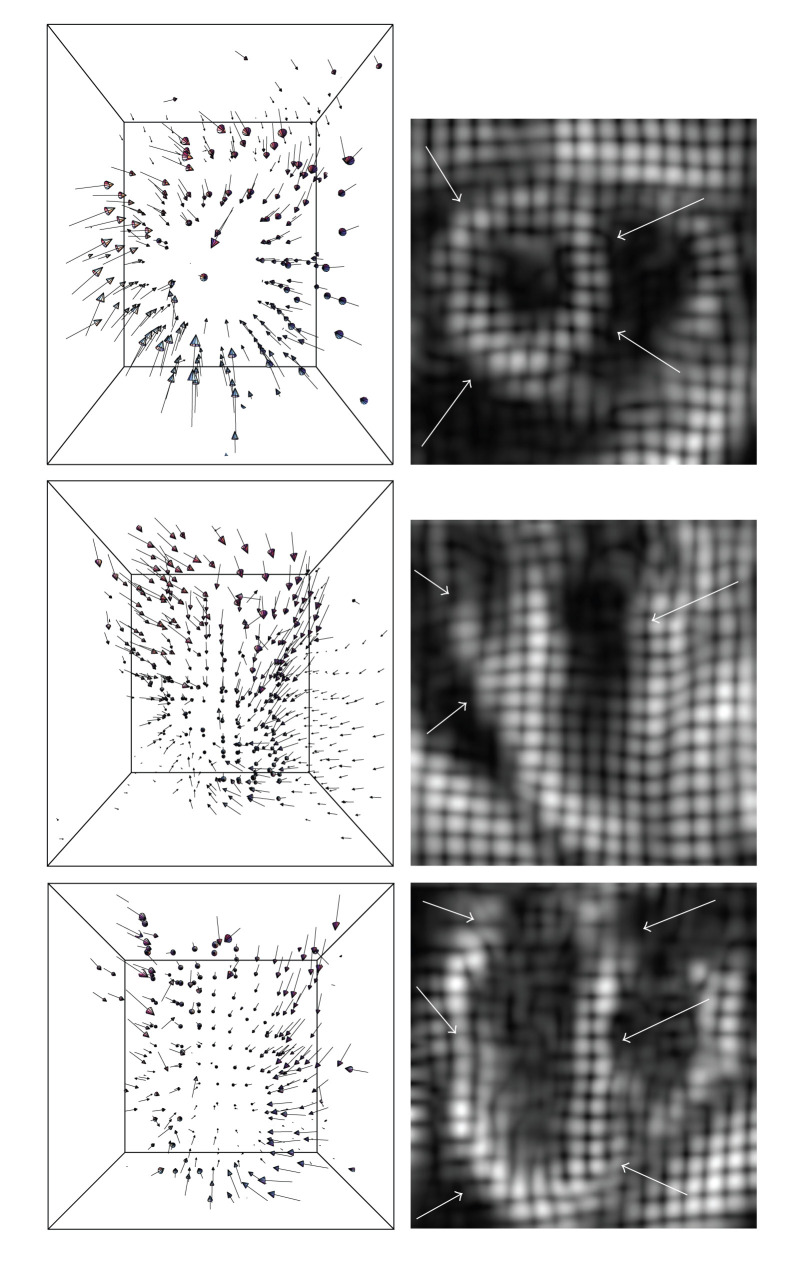
Three-dimensional velocity flow field of one frame of the left ventricle in phase of contraction (column 1) under 3 different views and the correspondent cross-sections of the cardiac image volume (column 2). In column 2, the left ventricle is highlighted by white arrows. Row 1 displays the short axis view, whereas rows 2 and 3 show the 2 long axis views. The retrieved 3-dimensional vectors illustrate with accuracy the cardiac motion behavior and overcome shortcomings typical of the 2-dimensional optic flow methods, such as through-plane motion detection.

The optic flow algorithm has also been applied on a real sequence of 23 tagged volume MR images representing a human beating heart. The images exhibit a resolution of 112 × 112 × 112 voxels and contained tags of 8 voxels wide. The velocity estimation is carried out at the tag crossings, the locations where critical points (maxima) are detected. The computation is carried out at a fixed scale of *s* = 3 voxels and also took roughly 5 to 10 minutes per frame. In Figure 7, we show the retrieved motion field for the cardiac data set investigated in the experiments. The images display the left ventricle in phase of contraction. After a qualitative inspection, we notice that the algorithm retrieves a critical point velocity in all three directions, providing valuable information for the quantitative analysis of the patient heart's dynamic behavior.

## 4. Discussion and Conclusion

 This paper investigates the 3D winding number as a efficient tool to retrieve and classify critical points in volume images. We provide a new formulation of the 3D winding number, simplifying the mathematics and implementation involved with respect to previous work [18]. We discuss the advantages of the proposed technique such as its ability to both locate and classify critical points. We carry out tests on three different real applications (ovarian follicle and neuron counting and cardiac motion estimation from tagged MRI). We finally discuss the experimental results, and we show their qualitative and quantitative reliability. 

In our applications, we highlight the usefulness of our algorithm in tedious and repetitive operations such as particle counting. The algorithm is able to find blobs and distinguish different cells located next to each other in all data sets. In order to carry out manual counting, the user may either count cells slice by slice or, to speed up the procedure, may perform a 3D projection of the slices and carry out manual counting. In this latest case, he may miss certain cells that are close but behind the ones located on the top. On the neuronal data set, for instance, our method detected 4 cells with roughly similar in-plane location (distance less than 3.6 *μ*m with respect to each other) but different height. 

In the experiment with the follicles and neurons, we highlight that our algorithm detects a similar amount of follicles and neurons as a trained echographer and neurobiologist, which is already a strong advantage of the proposed method. However, critical point detection has been carried out with a scale chosen globally. A critical point extraction performed at small scale might detect noisy grains (false positives). On the other hand, a critical point search carried out at too high scales may miss locations of follicles/neurons that present a smaller structure with respect to the other follicles/neurons in the data set. These problems might be avoided by choosing different scales for follicles/neurons with different sizes. In the future, we will carry out experiments in this direction. 

In the experiments, we assume that the cells have a roughly spherical shape. The neurons, however, have a roughly spherical head (the soma), connected to a tail (the axon). In this case, extremal points were sometimes found in the axons. The algorithm may, therefore, count twice the same cell, increasing the error of the final estimation (see Figure 4). A way to overcome this problem would be to take into account the geometry of the neuron and remove outcomes coming from the axon. In future research, we will tune the algorithm to this specific application.

So far, we have considered the winding number in the context of scalar images. However, other applications of the 3D winding number might be investigated such as detection of singularities in 3D vector fields [47]. These have been proved to be helpful in the visualization of 3D flow fields [48]. In the biomedical context, this could be applied to improve the visualization of blood flow. 

In the literature, as we already discussed, other critical point retrieval methodologies are known. Critical points estimation can be carried out by taking into account the local intensity [21], where the intensity of each voxel is compared with that of the respective 26 neighbors. In Section 3.2, we compared the performance of the 3D winding number algorithm with respect to that of the critical point detection method based on local intensity estimation. Both methods provided similar counting estimation. However, the intensity-based method is able to locate only maxima and minima, while the 3D winding number provides also information for saddle points. The 3D winding number algorithm is, therefore, preferable since it is able to characterize all types of critical points. In the future, we will carry out experiments on 3D saddle points detection, which have interesting applications in flow visualization [49, 50]. Finally, we will also compare the 3D winding number algorithm with other feature points detectors such as SIFT for 3D applications.

## Figures and Tables

**Table 1:** Index and winding number of critical points in 2D.

2D	Index	Winding number
Minimum	0	+2*π*
Saddle	1	−2*π*
Maximum	2	+2*π*

**Table 2:** Index and winding number of critical points in 3D.

3D	Index	Winding number
Minimum	0	+4*π*
1-saddle	1	−4*π*
2-saddle	2	+4*π*
Maximum	3	−4*π*

**Table 3:** Correspondence between the sign of the Hessian determinant and winding number for critical points in 3D.

3D	Sign (det *H*)	Winding number
Minimum	+	+4*π*
1-saddle	−	−4*π*
2-saddle	+	+4*π*
Maximum	−	−4*π*
